# Noise-induced hearing loss is correlated with alterations in the expression of GABA_B_ receptors and PKC gamma in the murine cochlear nucleus complex

**DOI:** 10.3389/fnana.2013.00025

**Published:** 2013-07-30

**Authors:** Zhen-Zhen Kou, Juan Qu, Dong-Liang Zhang, Hui Li, Yun-Qing Li

**Affiliations:** ^1^Department of Anatomy, Histology and Embryology, K. K. Leung Brain Research Centre, The Fourth Military Medical UniversityXi'an, China; ^2^Department of Otolaryngology, Xijing Hospital, The Fourth Military Medical UniversityXi'an, China

**Keywords:** CNC, GABA, GABA_B_R, PKCγ, GAD67-GFP knock-in mice

## Abstract

Noise overexposure may induce permanent noise-induced hearing loss (NIHL). The cochlear nucleus complex (CNC) is the entry point for sensory information in the central auditory system. Impairments in gamma-aminobutyric acid (GABA)—mediated synaptic transmission in the CNC have been implicated in the pathogenesis of auditory disorders. However, the role of protein kinase C (PKC) signaling pathway in GABAergic inhibition in the CNC in NIHL remains elusive. Thus, we investigated the alterations of glutamic acid decarboxylase 67 (GAD67, the chemical marker for GABA-containing neurons), PKC γ subunit (PKCγ) and GABA_B_ receptor (GABA_B_R) expression in the CNC using transgenic GAD67-green fluorescent protein (GFP) knock-in mice, BALB/c mice and C57 mice. Immunohistochemical results indicate that the GFP-labeled GABAergic neurons were distributed in the molecular layer (ML) and fusiform cell layer (FCL) of the dorsal cochlear nucleus (DCN). We found that 69.91% of the GFP-positive neurons in the DCN were immunopositive for both PKCγ and GABA_B_R1. The GAD67-positive terminals made contacts with PKCγ/GABA_B_R1 colocalized neurons. Then we measured the changes of auditory thresholds in mice after noise exposure for 2 weeks, and detected the GAD67, PKCγ, and GABA_B_R expression at mRNA and protein levels in the CNC. With noise over-exposure, there was a reduction in GABA_B_R accompanied by an increase in PKCγ expression, but no significant change in GAD67 expression. In summary, our results demonstrate that alterations in the expression of PKCγ and GABA_B_Rs may be involved in impairments in GABAergic inhibition within the CNC and the development of NIHL.

## Introduction

Human exposure to excessive noise has been linked to noise-induced hearing loss (NIHL) (Chung et al., 2005). Recent studies have suggested that noise overexposure potentially results in auditory threshold shifts in young adult animals via a series of neuroplastic changes in central auditory structures (Fritschy et al., 2008; Wang et al., 2009a).

The cochlear nucleus complex (CNC) occupies a pivotal position in the hierarchy of functional processes leading to convergence of auditory information. The CNC can be divided into the dorsal cochlear nucleus (DCN) and the ventral cochlear nucleus (VCN). The DCN receives peripheral afferents and sets up processing pathways into the inferior colliculus (IC). The DCN forms a layered structure: the molecular layer (ML), the fusiform cell layer (FCL), and the deep layer (DL) (Osen, 1969).

As shown in Figure 7, in the ML and FCL of the DCN, the glutamatergic fusiform cells receive inputs from auditory nerve afferents and parallel fibers, and project to the IC (Osen, 1969; Browner and Baruch, 1982). The inhibitory interneurons, GABA-containing cartwheel cells receive parallel fibers and make contacts on fusiform cells and other cartwheel cells (Nelson, 2004). The auditory nerve conducts auditory signals from the inner ear, and the parallel fibers integrate the multimodal sensory inputs, which encode proprioceptive information about the sound source and/or the suppression of body-generated sounds or vocal feedback (Nelson, 2004). It has been confirmed that noise overexposure alters synaptic transmission originating from both auditory nerve and parallel fibers within the DCN (Shore et al., 2008; Shore, 2011). Moreover, in auditory disorders, including hearing loss and tinnitus, dysfunction of the DCN, particularly in the neuronal circuitry of the ML and FCL has been linked to the disruption of GABAergic inhibition (Ling et al., 2005; Middleton et al., 2011).

Elucidating the intracellular mechanisms underlying long-term synaptic changes in the CNC is critical to understanding the development of auditory disorders (Chang et al., 2003). A group of key enzymes involved in intracellular signal transduction cascades are the family of phospholipid-dependent kinases, the protein kinases C (PKC). Although at least 10 isoenzymes have been described, the gamma subtype of PKC (PKCγ) is calcium-dependent and exclusively expressed in neurons in the central nervous system (CNS) (Tanaka and Nishizuka, 1994; Saito and Shirai, 2002; Ding et al., 2005). PKCγ is activated by diacylglycerol (DAG) and Ca^2+^ in the presence of phosphatidylserine. PKCγ has been demonstrated to play a significant role in the neuroplasticity of the auditory pathway, particularly in the DCN (Tanaka and Nishizuka, 1994; Saito and Shirai, 2002; Kou et al., 2011). However, little is known about the possible relationship between the PKCγ signaling pathway and GABAergic inhibition in the DCN, particularly in NIHL.

It is reported that gamma-aminobutyric acid is synthesized by two glutamic acid decarboxylases (GAD): GAD67 and GAD65 (Pinal and Tobin, 1998), which readily influence the cellular and vesicular GABA content (Murphy et al., 1998; Engel et al., 2001). Between the two isoforms, GAD67 is responsible for over 90% of basal GABA synthesis and is produced at limiting levels in the CNS (Asada et al., 1997; Chattopadhyaya et al., 2007). Because there is a lack of suitable reagents to positively identify GABAergic neurons, GAD67-GFP knock-in mice have been used to reveal many (if not most) GABAergic neurons in brain.

GABA mediates its inhibitory effects by activating GABA receptors, including GABAA receptors (GABA_A_Rs) and GABA_B_ receptors (GABA_B_Rs) (Jones et al., 1998; Kaupmann et al., 1998). It has been reported that in the CNC, GABA_A_R was unchanged after acoustic injury (Song and Messing, 2005; Dong et al., 2010). In contrast, NIHL has been reported to be associated with the reduced GABA_B_R-mediated GABAergic inhibition in IC and auditory cortex. However, there is little information regarding the expression of GABA_B_Rs in the DCN following NIHL (Szczepaniak and Moller, 1995; Aizawa and Eggermont, 2006).

To better understand how PKCγ in GABAergic inhibition might function within the CNC, we studied the distribution patterns of GAD67, PKCγ and GABA_B_Rs in the CNC, particularly in the DCN. Then we investigated whether GAD67, PKCγ and GABA_B_Rs were correlated with NIHL.

For this purpose, we first specified the exact localizations of GAD67, PKCγ, and GABA_B_Rs in the CNC. Because BALB/c mice and C57 mice are vulnerable to NIHL (Ohlemiller et al., 2000), then we used the two strains to detect the alterations of GAD67, PKCγ, and GABA_B_R expression at both mRNA and protein levels in NIHL.

## Methods

### Subjects

Because of the limited ability to identify the GABAergic neurons in brainstem with the use of antibodies against GABA or GAD (Sloviter and Nilaver, 1987; Sloviter et al., 2001) and GAD67 is responsible for over 90% of basal GABA synthesis in brain, we revealed the localization of GABAergic neurons in the CNC by employing the GAD67-GFP knock-in mice, in which the GAD67 mRNA is colocalized with GFP, and GFP is expressed in GABAergic neurons (Tamamaki et al., 2003; Huang et al., 2008; Han et al., 2010). The generation of GAD67-GFP knock-in mice has been reported previously and used widely for detecting GABAergic neurons (Tamamaki et al., 2003; May et al., 2008; Young and Sun, 2009; Chen et al., 2010; Han et al., 2010; Bang and Commons, 2012). In our studies, the mice were housed in standard conditions (12 h light/dark cycles) with water and food available *ad libitum*. Both young C57 and BALB/c mice are especially vulnerable to noise (Ohlemiller et al., 2000). In the present study, 6 young (2-month-old) male GAD67-GFP knock-in mice (C57 genetic background), 30 C57 mice (2-month-old) and 30 BALB/c mice (2-month-old) weighing 25–30 g were utilized. The Animal Care and Use Committees of the Fourth Military Medical University reviewed and approved all protocols.

### Immunohistochemistry

The mice were anesthetized and perfused for microscopy examination. Briefly, after deeply anaesthetizing by intraperitoneal injection of pentobarbital sodium (5 mg/100 g for mice), we perfused the mice with 80 ml of 4% (w/v) formaldehyde in 0.1 M PB as a fixative and post-fixed at 4°C for 4 h. The brainstems were obtained from 6 GAD67-GFP knock-in mice, 6 C57 mice and 6 BALB/c mice respectively, and then stored in 30% (w/v) sucrose solution in 0.05 M phosphate-buffered saline (PBS; pH 7.4) overnight at 4°C. The tissues were cut into transverse 20 μm thick serial sections in a cryostat (Leica CM1800, Germany), and used for immunofluoresence and Nissl staining (Hefti, 1986; Zhao et al., 2001).

The immunofluoresence protocol was performed as follows: the sections were blocked within 10% normal goat serum for 1 h. Triple-labeling fluorescent immunohistochemistry for GFP/GABA_B_R1/PKCγ was performed in GAD67-GFP knock-in mice. In order to identify the localization of GAD67-positive terminals specifically, we also did immunohistochemistry for GAD67/GABA_B_R1/PKCγ in C57 mice and BALB/c mice. The primary antibodies for triple-labeling fluorescent immunohistochemistry includes mouse antisera against GFP (1:500 dilution; Chemicon, Temecula, CA) or mouse antisera against GAD67 (1:500 dilution; Chemicon, Temecula, CA), guinea pig antisera against GABA_B_R1 (1:1000; Chemicon, Temecula, CA) and rabbit antisera against PKCγ (1:1000 dilution; Santa Cruz Biotechnology, Santa Cruz, CA). The sections were incubated with the primary antibodies for 48 h at 4°C. Then, after rinsing with 0.01 M PBS, the sections were incubated with species-specific secondary antibodies overnight in solutions containing: Alexa488-conjugated donkey anti-mouse IgG (1:500 dilution; Invitrogen, Carlsbad, CA), Alexa594-conjugated donkey anti-guinea pig IgG (1:500 dilution; Invitrogen, Carlsbad, CA) and Alexa647-conjugated donkey anti-rabbit IgG (1:500 dilution; Invitrogen, Carlsbad, CA). Finally, the sections were rinsed with 0.01 M PBS, mounted onto clean glass slides, air-dried and cover slipped with a mixture of 0.05 M PBS containing 50% (v/v) glycerin and 2.5% (w/v) triethylenediamine. The sections were observed under a confocal laser scanning microscope (FV-1000, Olympus, Japan), using appropriate laser beams and filters.

### Noise exposure

Noise exposure was performed in a double-walled sound attenuating room as described in previous studies (Ohlemiller et al., 1999; Han et al., 2009; Groschel et al., 2010). 24 BALB/c mice and 24 C57 mice were placed in two ventilated chambers, respectively. The mice had free access to food and water, and were acclimated to the environment for 1 week. Then 12 BALB/c mice and 12 C57 mice were exposed to a noise (4 kHz octave band, 110 dB SPL), 8 h per day for 14 days. The same number of mice was used as controls. Control mice were placed in the noise booth but not exposed to noise. In order to develop a noise-induced lesion, a RadioShack Supertweeter was attached to the top of the cages and driven by a power amplifier (Yamaha AX-500U, Japan) and a loudspeaker. The noise was amplified with noise levels being measured with a sound level meter (Bruel and Kjaer, type 2606). The noise level variation was less than 2 dB within the space available to the animals.

### Auditory brainstem response

Auditory brainstem response (ABR) was measured in the form of two blind tests at 1 day before noise exposure to determine the baseline and 14 days afterwards to determine auditory thresholds in each group as previously (Han et al., 2009; Kou et al., 2011; Lin et al., 2011; Qu et al., 2012). A previous study confirmed that after noise exposure for 14 days, the mice suffered from NIHL (Ohlemiller et al., 1999). Briefly, mice were anaesthetized with an injection of pentobarbital sodium (5 mg/100 g, i.p.) and the body temperatures were maintained at 37°C with a warm pad. The needle electrodes were inserted subcutaneously behind the pinna of the measured ear (active), at the vertex (reference) and in the back (ground). The stimulus signal was generated through Intelligent Hearing Systems (Bio-logic Systems, USA) which was controlled by computer and delivered by a Telephonics earphone (TDH 39, USA). Evoked responses to the ABR click stimuli were recorded and the thresholds were obtained for two ears. The stimulus signal was generated through an Intelligent Hearing Systems device (Bio-logic Systems, USA) controlled by a computer and delivered by an earphone. Stimuli (1-ms duration) were presented at a repetition rate of 10/s. The raw ABR waveforms were filtered from 100–3000 Hz bandwidth (Han et al., 2011, 2012; Li et al., 2011). Potentials were sent to a computer where the average waveform in response to 1024 sweeps was displayed. ABRs were obtained using descending intensity steps beginning at 80 dB sound pressure level (SPL) with steps of 5 dB and ending when visually discernible ABR waveforms could no longer be detected (Turner and Willott, 1998; Willott and Turner, 1999). Thresholds were then defined as the lowest intensity level at which a clear waveform was visible in the evoked trace.

### Real-time PCR

After deeply anesthetizing the mice, the fresh CNCs of mice in each group (*n* = 6) were harvested. Total RNA was extracted with Trizol reagent (Gibco BRL, USA) according to the manufacturer's instructions to synthesize single-stranded complementary DNA (cDNA). 2 μg of total RNA were subjected to reverse transcription reaction. The cDNA synthesis was performed using a synthesis kit (RR037A, TakaRa, Japan). The sequences of the primers for Real-Time PCR are listed in Figure 5A, in which glyceraldehyde 3-phosphate dehydrogenase (GAPDH) was selected as the housekeeping gene. For the amplification, 2 μ g cDNA was prepared with the SYBR^@^Premix Ex Taq™ (RR041A, TakaRa, Japan) and performed in a Real-Time PCR detection system (Applied Biosystems™, USA). The amplification protocols included 3 min at 95°C, denaturating at 95°C for 5 s by 40 cycles and annealing and extension at 60°C for 30 s. Calibrated and non-template controls were included in each assay. In each experiment, PCR reactions were done in triplicate and repeated three times in order to measure statistically valid results. Melting curve analysis was always performed at the end of each PCR assay. For the comparison of each gene, each relative mRNA expression was calculated with the following formula: 2^−deltadeltaCt^ (Livak and Schmittgen, 2001). The 2^−deltadeltaCt^ method is a valid way to analyze the relative changes in gene expression from real-time quantitative PCR (Lehrke et al., 2004; Pinal and Tobin, 2011; Su et al., 2011). The threshold cycle (C_t_) indicates the fractional cycle number at which the amount of amplified target reaches a fixed threshold. The deltadeltaCt defined as the difference in Ct values between experimental and control samples. For calibration, the control sample in each group was used and set to 100%.

### Western blotting

Because it is difficult to isolate the DCN from the CNC, and the results from immunohistochemistry indicated that the GFP/GABA_B_R1/PKCγ only distributed in the superficial layer of the DCN, not in the VCN, we harvested the whole fresh CNCs from anesthetized mice (*n* = 6 in each group). All procedures were performed on ice. Briefly, the materials were lysed in Eppendorf tube with 10 volumes of 50 mM Tris-HCl (pH 7.4), containing 300 mM NaCl, 1% Nonidet P-40, 10% Glycerol, 1 mM EDTA, 1 mM Na_3_VO_4_ and protease inhibitor cocktail (Roche, Switzerland). Then, the homogenized samples were centrifuged at 12,000 × g for 10 min at 4°C. Next, the lysate protein concentrations were determined with a BCA protein assay kit (Pierce, USA), and mixed with 5 × sodium dodecyl sulfate (SDS) sample buffer; boiled for 10 min. Equal samples of protein were electrophoresed by SDS-PAGE in 10% polyacrylamide gel. After electrophoretic transfer to nitrocellulose membrane, the blots were blocked with a blocking buffer (5% nonfat dry milk in TBS-T) for 2 h at room temperature and then incubated with primary antibodies diluted in 5% nonfat dry milk in TBS-T overnight at 4°C. The following primary antibodies were used: mouse antisera against GAD67, guinea pig antisera against GABA_B_R1, guinea pig antisera against GABA_B_R2 (1:500; Chemicon, Temecula, CA) and rabbit antisera against PKCγ. After incubation of the membrane with peroxidase-conjugated anti-rabbit secondary antibodies (Santa Cruz Biotechnology, Santa Cruz, CA) for 2 h at room temperature, the reaction products were visualized with enhanced chemiluminescence (Amersham Life Science, Amersham, UK).

### Statistical analysis

All data were subjected to statistical analysis using one-way ANOVA. Results are expressed as mean ± SEM by SPSS 13.0 (SPSS Inc.). The differences between groups were considered as statistically significant at a value of *p* < 0.05.

## Results

### Immunohistochemical detection of GFP, GAD67, GABA_B_R1s, and PKCγ in the CNC

Our studies indicate the localization of GABAergic neurons in the CNC by employing the GAD67-GFP knock-in mouse, in which GFP is specifically expressed in GABAergic neurons (Tamamaki et al., 2003; Li et al., 2005; Huang et al., 2008; Huo et al., 2009; Han et al., 2010).

**Figure 1:**
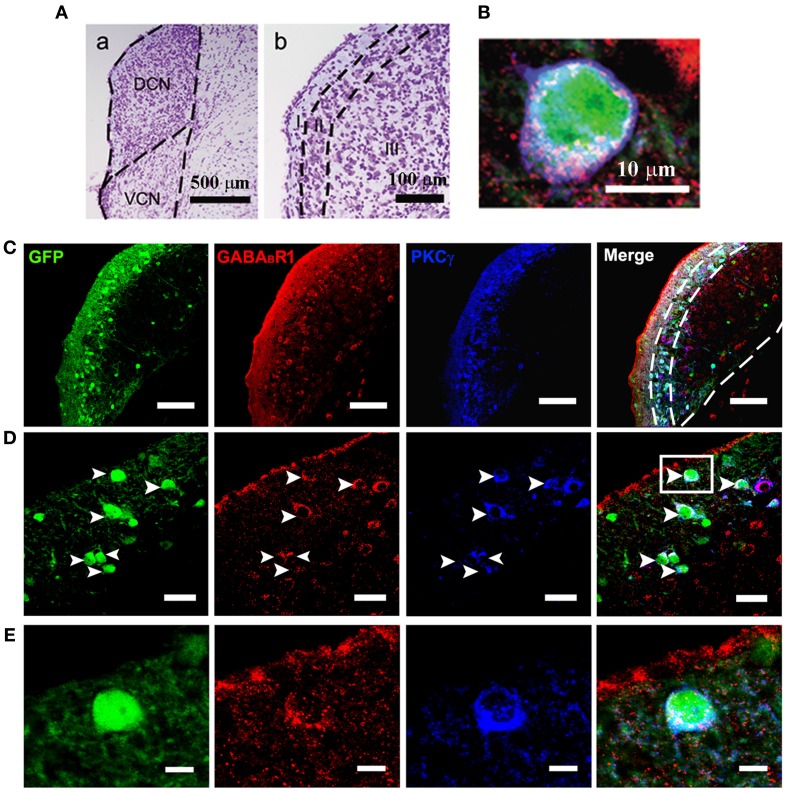
**Photomicrographs representing sections of the brainstem of GAD67-GFP knock-in mice in the DCN**. Nissl staining in the CNC **(A)** showing the dorsal part (DCN) and the ventral part (VCN) (a in **A**). The DCN organized into a layered structure (b in **A**): the molecular layer (I), the fusiform cell layers (II) and the deep layer (III). Triple-labeled neurons for GFP/GABA_B_R1/PKCγ were shown **(B)**. Fluorescent photomicrographs showing the distribution of the GFP-labeled (green), GABA_B_R1-positive (red) and PKCγ-positive (blue) cells in the molecular layer and fusiform cell layer of the DCN of the GAD67-GFP knock-in mice **(C)**. The arrowheads indicate the triple-labeled neurons for GFP/GABA_B_R1/PKCγ shown in **(D)**. The GABA_B_R1-immunoreactivities are located at the GFP-labeled GABAergic neurons which contain PKCγ (**E**, higher magnification areas, inserted panels in **D**). Scale bar: 500 μm in **(A**a**)**; 100 μm in **(A**b**)**; 10 μm in **(B)**; 100 μm in **(C)**; 30 μm in **(D)**; 10 μm in **(E)**.

**Figure 2:**
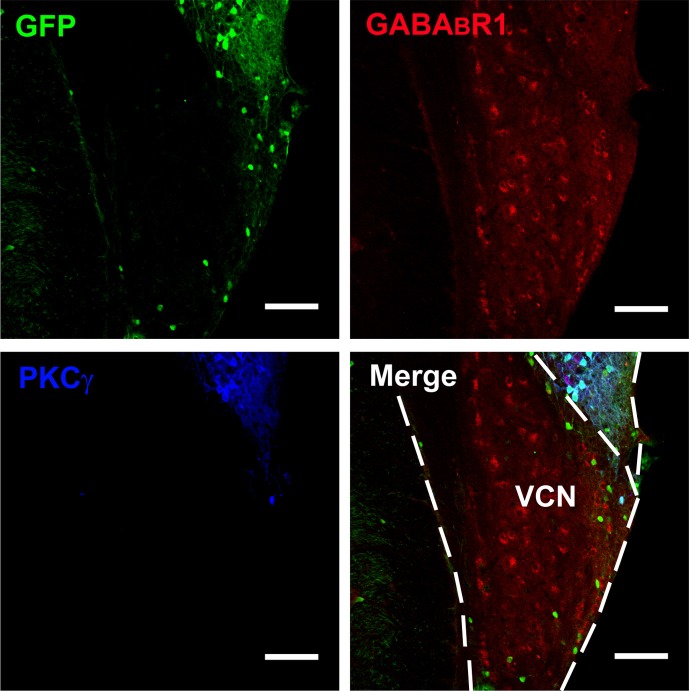
**Photomicrographs representing sections of the brainstem of GAD67-GFP knock-in mice in the VCN**. Fluorescent photomicrographs showing the distribution of GFP (green), GABA_B_R1-positive (red), and PKCγ-positive (blue) cells in the VCN of the GAD67-GFP knock-in mice. Triple-labeled neurons for GFP/GABA_B_R1/PKCγ were rare in the VCN. The bottom of the DCN can also be observed. Scale bar: 100 μm.

Confocal microscopy revealed an intense band of immunolabeling for the GFP-labeled neurons in the ML and FCL of the DCN (Figures 1A–C). The diameter of the GFP-labeled neurons is between 10 and 15 μm (Figures 1B,D). Occasionally, labeled neurons could be seen in the DL of the DCN (Figures 1A,C). In the VCN, the GFP-positive neurons were virtually absent (Figure 2).

It is believed that heterodimeric assemblies of GABA_B_R1 and GABA_B_R2 subunits are required to form full functional receptors (Jones et al., 1998; Kaupmann et al., 1998; White et al., 1998). It has been confirmed that GABA_B_R1 is colocalized with GABA_B_R2 in brain, including the CNC (Lujan et al., 2004). Thus, the antibody used in the present study was a useful tool for detecting heteromeric assembly for the functional GABA_B_Rs. In the present study, the immunoreactivities for GABA_B_R1 were scattered throughout the CNC, including both DCN and VCN (Figures 1, 2). However, the immunolabeling for GABA_B_R1 was most obvious in the ML and FCL of the DCN and gradually decreased to the DL (Figures 1A,C). In the VCN, the staining for GABA_B_R1-positive neurons was much weaker than in the DCN (Figure 2). We found that 53.81% of GABA_B_R1-positive neurons were GFP/GABA_B_R1/PKCγ triple-labeled neurons (Table 1). In addition, the GABA_B_R1-positive terminals were observed as punctate labeling in the DCN (Figures 1C,D).

**Table 1:** **The numbers of neurons immunopositive for GFP, GABA_B_R1, PKCγ, and triple-labeled neurons in CNC of the GAD67-GFP transgenic mice**.

	**CNC**
(1) GFP^+^ neuron (mean ± S.E.M.)	1243 ± 57.15
(2) GABA_B_R1^+^ neuron (mean ± S.E.M.)	1615 ± 27.28
(3) PKCγ^+^ neuron (mean ± S.E.M.)	1011 ± 23.34
(4) GFP/PKCγ (mean ± S.E.M.)	902 ± 16.15
(5) GFP/GABA_B_R1 (mean ± S.E.M.)	1124 ± 15.26
(6) GFP/PKCγ/GABA_B_R1 (mean ± S.E.M.)	869 ± 25.69
(7) (4)/(1) × 100%	72.57%
(8) (5)/(1) × 100%	90.43%
(7) (6)/(1) × 100	69.91%
(8) (6)/(2) × 100	53.81%
(9) (6)/(3) × 100	85.95%

*Counts in mouse were made by using ten sections from a series of every fourth serial section of 20 μm thickness*.

Our previous work revealed the specific distribution pattern of PKCγ in the mouse DCN (Kou et al., 2011). The PKCγ-positive cells were abundant in the DCN with moderate to strong staining in the ML and FCL, demonstrating an overlapping pattern with the GFP-labeled neurons (Figures 1A,C). The immunoreactivities for PKCγ were detected in the cell bodies and the processes of neurons (Figures 1B,D,E). We found that 85.95% of PKCγ-positive neurons were GFP/GABA_B_R1/PKCγ triple-labeled neurons (Table 1).

In GAD67-GFP knock-in mice, strong coexpression of GABA_B_R1 and PKCγ in the GFP-labeled neurons was observed in the ML and FCL of the DCN (Figure 1). We found that 90.43 and 72.57% of the GFP-labeled neurons were also immunopositive for GABA_B_R1 and PKCγ, respectively. GFP/GABA_B_R1/PKCγ triple-labeled neurons accounted for 69.91% of GFP-labeled neurons (Table 1). The triple-labeled neurons for GFP/GABA_B_R1/PKCγ were observed in small- to medium-sized neurons, with diameters were between 10 and 15 μm (Figures 1B,E).

**Figure 3:**
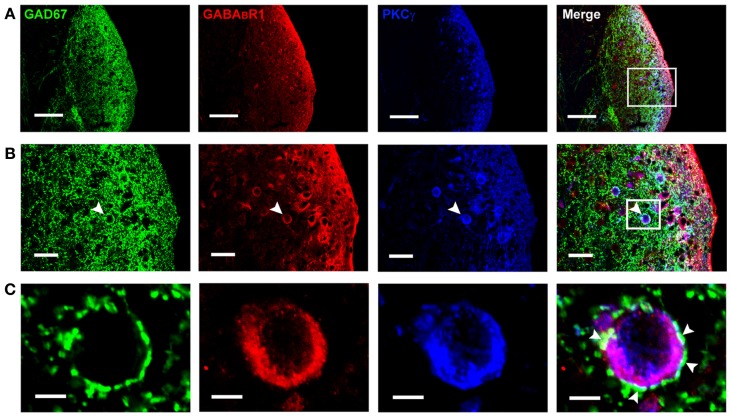
**Photomicrographs representing sections of the brainstem of C57 mice in the DCN**. Fluorescent images showing the GAD67-positive puncta are distributed densely in the molecular layer and fusiform cell layer of the DCN **(A)**. In **(B)**, the arrowheads indicate the triple-labeled neuron for GAD67/GABA_B_R1/PKCγ (higher magnification areas, inserted panels in **A**). In **(C)**, The arrowheads indicate that the GAD67-positive terminals made connections on the double-labeled neurons (higher magnification areas, inserted panels in **B**). Scale bar: 100 μm in **(A)**; 30 μm in **(B)**; 10 μm in **(C)**.

By using GAD67-GFP transgenic mice, we could observe the cell bodies of the GFP-labeled neurons, but it is difficult to define whether the GAD67-positive terminals are associated with the cell bodies. So we used the GAD67 antibody to recognize the GAD67-positive terminals in wild-type C57 and BALB/c mice, in order to determine the distribution of GABAergic terminals in the DCN. We found that GAD67-positive terminals were densely distributed in the ML and FCL of the DCN in both C57 (Figure 3A) and BALB/c mice (data not shown). The GAD67/GABA_B_R1/PKCγ triple-labeling experiments showed that a subpopulation of GAD67-positive puncta were associated with the cell bodies and the processes of the neurons colocalized with GABA_B_R1s and PKCγ (Figures 3B,C).

### Auditory threshold shifts after noise exposures

**Figure 4:**
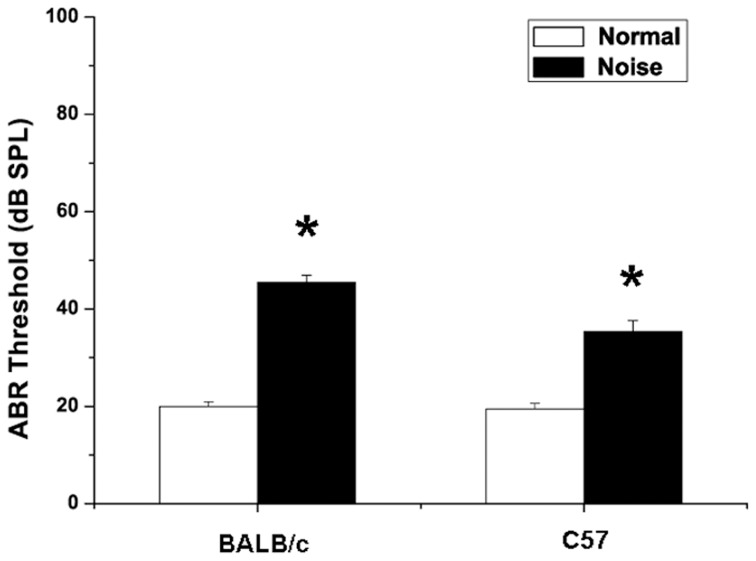
**Changes in auditory brainstem response of BALB/c mice and C57 mice after noise exposure**. Normal group: mice received no treatment and placed in the noise booth but not exposed to noise. Noise group: mice were exposed to 4-kHz octave-band noise at 110 dB SPL for 8 h per day for 14 consecutive day. ^*^P < 0.05.

There was no significant difference between the thresholds of ABR before noise exposure in groups, and hearing levels were essentially equivalent (18.04 ± 0.68 dB SPL in C57 mice, 19.35 ± 0.85 dB SPL in BALB/c mice, *p* > 0.05). NIHL was assessed on day 14 following noise exposure. As shown in Figure 4, there were elevations of ABR thresholds in noise-treated C57 mice (*p* < 0.05) and BALB/c mice (*p* < 0.05). In BALB/c mice exposed to noise, the hearing threshold increased significantly to 45.5 ± 1.4 dB SPL (*p* < 0.05). Compared with controls, the hearing threshold in C57 mice increased as well, being 35.4 ± 2.3 dB SPL after noise exposure (*p* < 0.05).

### Noise-induced alterations of GAD67, GABA_B_Rs, and PKCγ expression at mRNA level

To examine whether GAD67, GABA_B_R and PKCγ mRNA levels are changed after noise stimulation, the mRNA expression were measured by Real-Time PCR. The transcript levels of them were normalized to GAPDH. The 2^−deltadeltaCt^ analysis was adopted to quantify the relative changes of target gene expression (Livak and Schmittgen, 2001).

**Figure 5:**
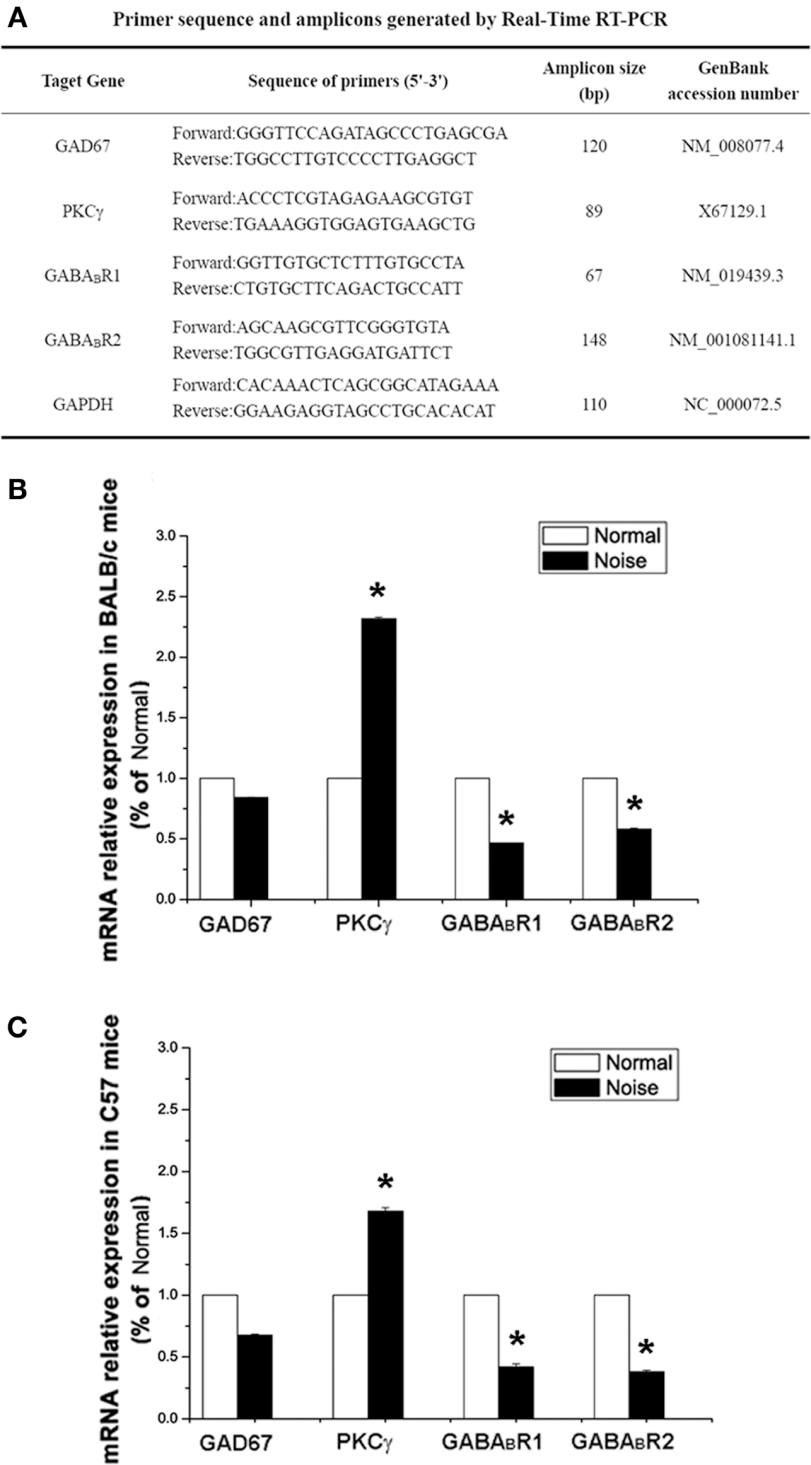
**The alterations of GAD67, GABA_B_R1, GABA_B_R2 and PKCγat mRNA levels after noise exposure in BALB/c mice and C57 mice**. **(A)** Sequences of specific primers and associated amplicon lengths for Real-Time PCR. **(B)** The noise-induced changes of GAD67, GABA_B_R1, GABA_B_R2, and PKCγ mRNA levels in BALB/c mice of each group. The sample of control group was set as 100%. **(C)** The noise-induced changes of GAD67, GABA_B_R1, GABA_B_R2, and PKCγ mRNA levels in C57 mice. The sample of control group was set as 100%. ^*^P < 0.05.

As demonstrated in Figures 5B,C, after noise exposure, there were slight decreases in GAD67 mRNA expression in both BALB/c and C57 strains, but no significant difference (*p* > 0.05). However, we found a decrease in GABA_B_R mRNA expression, showing significant differences between the control group and the noise exposure group in both BALB/c mice (*p* < 0.05) and C57 mice (*p* < 0.05). A prominent increase in PKCγ mRNA expression was found in the noise exposure group. The data showed that the PKCγ mRNA expression increased by 2.3-fold compared with controls in BALB/c mice (Figure 5B, *p* < 0.05). In C57 mice, the PKCγ mRNA expression also showed an increase of 1.7-fold compared to the controls (Figure 5C, *p* < 0.05). Taken together, these results indicate that noise exposure induced a decrease in GABA_B_R mRNA and increases in PKCγ mRNA in the CNC, but GAD67 mRNA was unaffected.

### Noise-induced alterations of GAD67, GABA_B_R, and PKCγ expression at protein level

**Figure 6:**
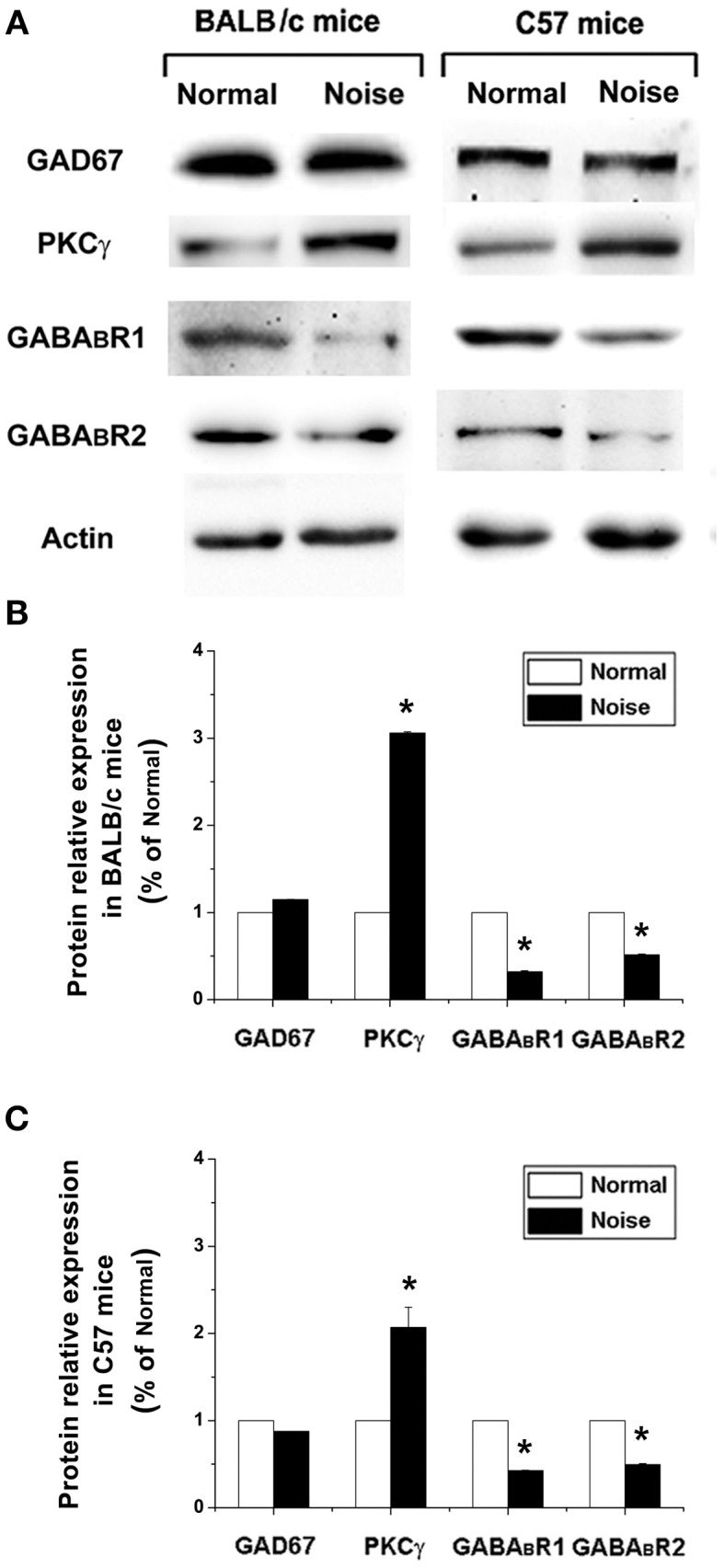
**The alterations of GAD67, GABA_B_R1, GABA_B_R2, and PKCγ at protein levels after noise exposure in BALB/c mice and C57 mice**. **(A)** GAD67, GABA_B_R1, GABA_B_R2, and PKCγ protein levels detected by Western blotting. **(B)** The noise-induced changes of GAD67, GABA_B_R1, GABA_B_R2, and PKCγ expressions in BALB/c mice. **(C)** The noise-induced changes of GAD67, GABA_B_R1, GABA_B_R2, and PKCγ expressions in C57 mice. ^*^P < 0.05.

We next examined whether GAD67, GABA_B_R, and PKCγ protein levels were also changed after noise exposure. To this end, we performed Western Blotting and quantified GAD67, GABA_B_R, and PKCγ protein levels at the same time points as mRNA detection. The results were normalized to the densitometry values of β-actin, the relative protein levels were presented in Figure 6A. As for the mRNA analysis, a one-way ANOVA indicate that there was no significant effect of noise on GAD67 protein levels in BALB/c mice (*p* > 0.05) and C57 mice (*p* > 0.05). However, in GABA_B_R protein expression, data obtained here indicate that there was a significant decrease in the noise exposure group (Figure 6, *p* < 0.05). In BALB/c mice, the PKCγ protein level increased significantly after noise exposure (Figures 6A,B, *p* < 0.05). In C57 strain, the expression of PKCγ was significantly elevated in the noise exposure group (Figures 6A,C, *p* < 0.05). These results suggest that after noise treatment, the alterations of GABA_B_R and PKCγ at protein levels were statistically significant in the CNC of BALB/c mice and C57 mice.

## Discussion

Our results suggest that a large subpopulation of GABAergic neurons in the ML and FCL of the DCN coexpress GABA_B_R1s and PKCγ. The GAD67-positive terminals were attached to the GABA_B_R1/PKCγ double-labeled neurons. Moreover, after noise exposure, both C57 mice and BALB/c mice exhibited obvious hearing deficits and these functional effects were accompanied with changes in GABA_B_R and PKCγ expression in the CNC. Thus, the alterations of GABA_B_Rs and PKCγ in GABAergic inhibition in the DCN might be associated with NIHL.

### GABAergic inhibition in NIHL

It is noteworthy that disruption in GABAergic inhibition within the DCN has been implicated in the development of hearing impairment in rodents (Ling et al., 2005; Wang et al., 2009a). In addition, another important inhibitory neurotransmitter, glycine and its receptor (GlyR) have been reported in the DCN (Rubio and Juiz, 2004). In age-related hearing loss or unilateral cochlear ablation, decreased glycine levels and the reduction of the total number of GlyR binding sites were found in the DCN, suggesting the down-regulation of glycinergic inhibition is also associated with hearing loss in the DCN (Willott et al., 1997; Potashner et al., 2000; Wang et al., 2009b).

The suppression of GABAergic inhibition in hearing loss could be attributed to two reasons: decreased GABA synthesis and release or down-regulation of its receptors. In our study, results from Real-Time PCR and Western Blotting analysis showed that after noise stimulation, the GABA_B_R expression decreased at both mRNA and protein levels. In contrast, the endogenous promoter of GAD67, a specific marker for GABAergic neurons remained unchanged in hearing impaired mice. Thus, our data suggest that rather than decreased GABA synthesis and release in the CNC, the preferential decrease in GABA_B_R expression may reflect a selective loss of GABAergic inhibition in NIHL.

### Decreased GABA_B_Rs after noise injury in NIHL

Previous results indicate that GABA_B_Rs are expressed in both GABAergic cartwheel cells and glutamatergic fusiform cells in the DCN, suggesting that GABA_B_Rs may participate in both inhibitory and excitatory effects in the DCN (Lujan et al., 2004; Irie and Ohmori, 2008). Moreover, the excitability of both cartwheel and fusiform cells was shown to be enhanced in the DCN following noise trauma (Brozoski et al., 2002; Chang et al., 2002).

**Figure 7:**
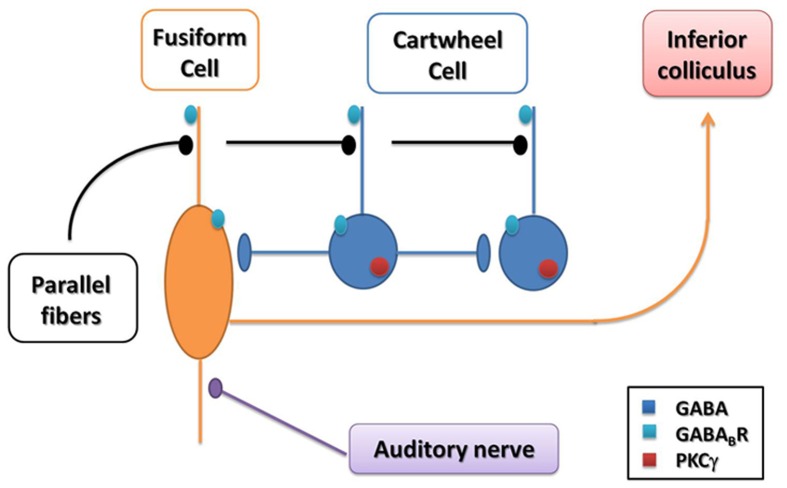
**Summary diagram of the circuitry in the DCN**. The GABA/GABA_B_R/PKCγ triple-labeled neurons are distributed in the molecular layer and fusiform cell layer. Most of these neurons are presumed to be cartwheel cells, which receive input from parallel fibers. As inhibitory interneurons, their GABAergic terminals were attached to the other cartwheel cells and fusiform cells. The glutamatergic fusiform cells, which might also express GABA_B_Rs, receiving inputs from parallel fibers and auditory nerve afferents, finally project to the inferior colliculus (IC). The noise stimulation could activate both cartwheel cells and fusiform cells. The activation of cartwheel cells could be attributed to the decreased GABA_B_Rs, leading to the disinhibition among the GABA-containing cartwheel cells. The noise injury also increases PKCγ in these activated cartwheel cells. In the glutamatergic fusiform cells expressing GABA_B_Rs, because of the reduction of GABA_B_Rs, the loss of presynaptic and postsynaptic inhibition of glutamate activation might promote glutamate neurotransmission in NIHL.

The morphological features of cartwheel cells have been identified in the DCN (Osen, 1969; Wouterlood and Mugnaini, 1984). Firstly, cartwheel cells distribute at the ML and FCL. Secondly, cartwheel cells (10–20 μm in mean cell body diameter) are small- to medium size, and have large primary dendrites (Wouterlood and Mugnaini, 1984). Thirdly, the axons of cartwheel cells terminate over a relatively restricted area and make contact with other cartwheel neurons as well as fusiform neurons (Berrebi and Mugnaini, 1991). Fourthly, the cartwheel cells are thought to be inhibitory because they are strongly labeled by antisera against GAD, GABA, and glycine (Mugnaini, 1985). Finally, unlike other types of cells in the DCN, it has been demonstrated that cartwheel cells express PKCγ specifically. (Garcia and Harlan, 1997). According to these features, the cartwheel cell could be identified (Idrizbegovic et al., 2004; Kou et al., 2011). In the present study, the triple-labeled neurons for GFP/GABA_B_R1/PKCγ (10–15 μm in mean cell body diameter), in addition to the morphology and the distribution in the DCN, suggest that they might correspond to cartwheel cells in the ML and FCL of the DCN (Figure 7).

Cartwheel cells send GABAergic axons and contact other cartwheel neurons and fusiform cells. Our results also suggest that the GAD67-positive terminals were attached to the GABA_B_R1/PKCγ double-labeled neurons. Therefore, we presumed that after noise stimulation, the increased activity of cartwheel cells could be partially explained by disinhibition, through our observed decrease in GABA_B_R expression.

Because GABA_B_Rs were also located at both presynaptic and postsynaptic sites of glutamatergic fusiform cells in the DCN (Lujan et al., 2004). At the presynaptic site, electrophysiological investigations have shown that GABA_B_R mediates presynaptic inhibition of excitatory neurotransmission through G protein-mediated modulation of presynaptic Ca^2+^ channels, or lowering cyclic AMP and then blocking the stimulatory effect of increased Ca^2+^ on vesicle recruitment (Isaacson, 1998; Sakaba and Neher, 2003). Importantly, noise exposure has also been implicated in stimulating glutamate activation (Isaacson, 1998; Groschel et al., 2011; Shore, 2011; Dehmel et al., 2012). Thus, the downregulation of presynaptic GABA_B_Rs at fusiform glutamatergic terminals may result in the disinhibition of glutamate release in the DCN, possibly contributing to NIHL.

Previous results suggest that GABA “spillover” from neighboring sites may represent a possible source of GABA in the activation of GABA_B_Rs in the DCN (Lujan et al., 2004). GABA_B_Rs located at a distance from GABA releasing sites could be activated by GABA “spillover” has also been confirmed in the hippocampus and the cerebellum (Lopez-Bendito et al., 2002, 2004). At postsynaptic sites, GABA_B_R could activate inwardly rectifying K^+^ channels (GIRK/Kir3) via G protein β/γ subunits (Otmakhova and Lisman, 2004). The GIRK channels mediate slow hyperpolarizing currents which are important for shunting excitatory synaptic currents and can control glutamate receptor activation (Otmakhova and Lisman, 2004). Therefore, GABA_B_Rs located at the postsynaptic sites of glutamatergic fusiform cells (Figure 7), might fulfill a control function on glutamate excitatory transmission by GABA “spilling over” from nearby GABAergic circuitry. Moreover, in the present study, the reduced GABA_B_Rs might suggest a role in the decreased GABAergic inhibition from the GABAergic terminals on the fusiform cells in NIHL (Figure 7).

It has been reported that GABA_A_Rs are distributed in the CNC. However, after acoustic trauma, there was no significant difference in GABA_A_R mRNA expression in the CNC between the control mice and the hearing impaired mice (Song and Messing, 2005; Dong et al., 2010). Therefore, alterations in the expression of GABA_B_Rs may play a more important role than changes in GABA_A_R expression in the development of hearing loss.

### Increased PKCγ after noise injury in NIHL

As an important second-messenger-activated protein kinase, it is accepted that PKCγ plays an important role in brain, including the DCN (Colombo and Gallagher, 2002; Rossi et al., 2005; Kou et al., 2011) In this study, 85.95% PKCγ-positive cells were found to coexpress GAD67 and GABA_B_R1s, suggesting a possible role in GABAergic inhibition.

PKCγ has been suggested to be an injury-activated intracellular modulator that promotes neuronal activity in various neuroprotective signal pathways (Colombo and Gallagher, 2002; Hayashi et al., 2005; Rossi et al., 2005). Noise stimulation could increase the activity of GABA-containing cartwheel cells in the DCN (Brozoski et al., 2002; Chang et al., 2002). In addition, PKCγ is specifically expressed in cartwheel cells (Garcia and Harlan, 1997). In our study, we found that 85.95% of PKCγ-positive neurons coexpressed GAD67 and GABA_B_R1s, indicating that the selective increase in PKCγ expression may serve as a noise injury-induced intracellular modulator in the subpopulation of GABA/GABA_B_R1/PKCγ cartwheel cells in the DCN (Figure 7).

In addition, we noted that there was no statistically significant difference in GAD67 expression at both mRNA and protein levels. Although the neuroprotective role of PKCγ has been reported, after noise stimulation, whether the unchanged GAD67 expression was due to the activated PKCγ or the feedback effects of the reduced GABA_B_Rs needs further investigation in NIHL.

Although the triple-labeled neurons contained GAD67, GABA_B_R1s, and PKCγ distributed densely in the DCN, there were also several neurons which expressed PKCγ, GABA_B_R1s, or GAD67 but were not triple-labeled. This might be explained by the following reasons. First, a group of fusiform cells might express GABA_B_Rs. Second, as well as the cartwheel cells (the diameter of cell bodies is between 10 and 20 μm), the stellate cells (the diameter of cell bodies is usually less than 10 μm) could contain GABA (Mugnaini, 1985). Third, the tissue treatment or the process of triple-labeling immunohistochemistry might also affect the detection levels of the antibodies.

## Conclusion

In summary, our results focused on the CNC of mice subjected to the influence of noise exposure, particularly in the GABAergic neurons in the ML and FCL of the DCN. Because disruption of GABAergic inhibition in the DCN has been confirmed in auditory disorders, particularly in hearing loss (Ling et al., 2005; Middleton et al., 2011), the decreased expression of GABA_B_Rs may, at least in part, play a role in altered inhibitory processing in NIHL. Although we assume that the unchanged GAD67 may be associated with the neuroprotective role of PKCγ, investigating the interactions of GABA, PKCγ, and GABA_B_R in the CNC in greater detail should be informative in developing a better understanding of the pathogenesis of NIHL.

### Conflict of interest statement

The authors declare that the research was conducted in the absence of any commercial or financial relationships that could be construed as a potential conflict of interest.
